# The experiences of young adults attempting to quit e-cigarettes: A mixed-methods analysis

**DOI:** 10.18332/tpc/202831

**Published:** 2025-04-16

**Authors:** Tyler Merreighn, Jennifer C. Veilleux, Eric D. Schisler, Mufazzela Tabassum, Page D. Dobbs

**Affiliations:** 1 Department of Health, Human Performance and Recreation, University of Arkansas, Fayetteville, United States; 2 Center for Public Health and Technology, University of Arkansas, Fayetteville, United States; 3 Department of Psychological Science, University of Arkansas, Fayetteville, United States; 4 Department of Healthcare Studies, Salem State University, Massachusetts, United States

**Keywords:** e-cigarette, tobacco, quitting, distress, mixed methods

## Abstract

**INTRODUCTION:**

Young adult users of e-cigarettes have expressed intention to quit using these products. The purpose of this study was to explore the experiences of young adult e-cigarette users with trying to quit e-cigarettes.

**METHODS:**

Using an explanatory sequential mixed-methods design, a convenience sample of young adults living in the US in 2021 who had used e-cigarettes in the past 30 days completed a cross-sectional survey (n=592), and then a subsample of participants (n=25) completed a follow-up Zoom interview. Relationships between e-cigarette dependence and quitting were examined along with differences between motives for use, abstinence experiences, and stress intolerance of those who had and had not tried to quit e-cigarettes, and associations between quitting e-cigarettes and covariates. Interviewees discussed quit attempts and reasons to quit e-cigarettes. All interviews were transcribed verbatim, data were coded, and emergent themes were compared to quantitative results.

**RESULTS:**

Most participants (73.5%) had attempted to quit using e-cigarettes. Variables associated with quit attempts included past cigarette smoking (AOR=1.72; 95% CI: 1.06–2.81), cognitive coping (AOR=0.056; 95% CI: 0.42–0.75), loss of control (AOR=1.45; 95% CI: 1.08–1.94), and cue exposure (AOR=1.40; 95% CI: 1.12–1.76). Increased e-cigarette dependence was associated with more e-cigarette quit attempts (p<0.001) and shorter periods of abstinence from using e-cigarettes (p<0.001). Emergent interview themes described social (e.g. environments), emotional (e.g. using when distressed), and physical (e.g. withdrawal symptoms, including headaches and shaking) barriers to quitting e-cigarettes.

**CONCLUSIONS:**

Tobacco prevention messages, research, and cessation services should consider that young adults may experience co-occurring distress during e-cigarette quit attempts.

## INTRODUCTION

Electronic cigarette (e-cigarette) use in the US has increased among youth and young adults over the past decade ^1^. In 2019, reported e-cigarette use among adults aged ≥18 years was highest among those aged 18–24 years (9.3%) ^2^. Although the long-term health effects of e-cigarettes are still under exploration, initial concerns include the effect of nicotine on brain development ^3^, adverse respiratory symptoms ^4^, potential for nicotine addiction ^5^, and transitions to cigarette initiation and use ^6^. Young adults who use e-cigarettes are aware of their nicotine addiction (compulsive behavior that includes seeking out and use of drugs), but some may minimize this physiological dependence (withdrawal symptoms during abstinence from drug use), believing such addiction to be normal ^7^. Young adults (18–24 years) have expressed an interest in and expectation of quitting e-cigarettes ^8^.

Nationally representative samples report that 44.5% of US youth (12–17 years) who use e-cigarettes have seriously considered quitting, and about a quarter have attempted to quit at least once ^9^. Reasons youth and young adults (13–24 years) reported for wanting to quit using e-cigarettes include money, freedom from addiction, influence from social referents, and concerns about physical or mental performance (e.g. sports, academic, vocal, cognitive/attention, and sexual) ^10^. Understanding factors that facilitate and barriers that inhibit successful e-cigarette quit attempts are valuable given that young populations that initiate nicotine use with e-cigarettes may become dependent cigarette smokers ^6^, and the global economic burden of smoking has been estimated at $1436 billion ^11^. Further, about 80% of those who smoke before 18 will subsequently become lifetime smokers before turning 21 years ^12^. Thus, quitting nicotine products is recommended for all ages ^13^.

Research suggests exclusive e-cigarette users and dual users (those who use both cigarette and e-cigarettes) believe it is important to quit using all nicotine products ^14^. To address this, cessation programs have been developed for youth and young adults attempting to quit using e-cigarettes ^15^. Research suggests US youth who experience psychological distress are more likely to use disposable e-cigarettes ^16^, and in a randomized control trial, e-cigarettes users with high levels of anxiety were significantly less likely to successfully quit using e-cigarettes than those with less anxiety ^17^. What is unknown is how well young adults who use e-cigarettes tolerate distress and if there are differences between distress intolerance and those who have attempted to quit using e-cigarettes. Understanding young people’s experiences with past e-cigarette quit attempts and their ability to tolerate distress may help those developing and modifying cessation strategies to improve quitting attempts among young adult e-cigarette users. Smokers report greater problems tolerating and withstanding their distress than non-smokers ^18^. Considering e-cigarette use is linked to increased depression and anxiety symptoms ^19^, it is possible that people may struggle to quit e-cigarettes if they use these products to cope with or avoid distress. Thus, the purpose of this study was to examine relationships between young adult e-cigarettes users’ distress intolerance, motivations for use, and intolerance of abstinence based on their prior quitting behaviors.

## METHODS

### Design

The current explanatory sequential mixed-methods study first employed a cross-sectional survey (n=592) and then used a follow-up interview with a subsample of participants (n=25). Mixed-methods research emphasizes the priority (distinguished by capitalization) of one methodology, and benefits of this research approach include using the other methodology to complement or enhance the findings of the first ^20^. For the current research design, the priority emphasized the quantitative findings that were further explained by the subsequent qualitative results.

### Procedures and participants

Participants for both phases were recruited by the survey platform Prolific, during October 2021. Prolific is a survey medium that recruits people to complete surveys for payment, and users complete screening measures that allow for selection of populations. Convenience sampling was employed, using Prolific’s feature to only recruit those who meet eligibility criteria. Eligibility included being aged 18–29 years, living in the US, and used e-cigarettes regularly (used tobacco products and e-cigarettes, previously smoked and only use e-cigarettes, and only ever use e-cigarettes). Participants were paid $3.96 to complete the 25-minute survey.

Next, all survey participants were invited to complete a follow-up virtual interview. The first 25 participants who enrolled were scheduled for an interview by providing the participants with a link to a virtual integration software (Calendly). They were then provided with a Zoom link and reminders one day and one hour before the interview. Participants were asked to create an alias name when signing up for the interview, and their Zoom title was changed to their participant number before the interviews were recorded. Following the 60-minute interview, participants were paid $50 directly through Prolific. Saturation (where themes affirmed those from prior interviews) was met with 20 interviews, and five more interviews were conducted to ensure no new themes emerged. All procedures were approved by the University of Arkansas Institutional Review Board.

### Measures

In addition to demographic items (gender, race/ethnicity, age, education level, marital status), participants were asked about past smoking behaviors. Smoking status (dual use of cigarettes and e-cigarettes, past smoker, and never smoker) was classified by responses to two items: ever smoking (yes/no) and past 30-day smoking (yes/no). Those who had ever smoked a cigarette (even once in their lifetime) but not in the past 30 days were considered past smokers, those who smoked in the past 30 days were dual users, and those who had never smoked a cigarette were never smokers. E-cigarette use was measured by asking participants what type of device they used in the past 30-days with pictures depicted next to the question of popular devices at the time data were collected [device options included vape pens, mod or mech mod rechargeable devices, box mod devices, JUUL, other (non-JUUL) pod devices, Puff Bar, other (non-Puff Bar) disposable devices)]. For this study, the type of device was only used to confirm past-30-day use of all users. Further, e-cigarettes were defined for participants as ‘electronic nicotine products’ to clarify between vaping devices used for cannabis. E-cigarette dependence was measured using the Penn State E-cigarette Dependence Index ^21^. Scores for this 10-item measure range 0–20: no (0–3), low (4–8), moderate (9–12), and high dependence (13–20).

Given the timeframe data were collected, participants were asked: ‘Over the past year (during the coronavirus pandemic), do you believe you have increased or decreased the number of e-cigarettes used per day?’. Response options included: decreased, stable, or increased. Current depressive and anxiety symptoms were assessed using a two-item Patient Health Questionnaire (PHQ-2) and two-item Generalized Anxiety Disorder (GAD-2), respectively ^22,23^.

Five items were used to measure e-cigarette quitting. Participants were asked if they had ever tried to quit using e-cigarettes (yes/no response), and if they had tried to quit, they were asked the number of times they had attempted to quit or cut down on their use of e-cigarettes. Responses included: 1, 2, or ≥3. Next, participants were asked how long it had been since their last quit attempt; responses included within a week, month, last six months, and more than six months. Responses were collapsed into the past month (30 days), the last six months, and more than six months ago. Participants reported length of their last quit attempt (i.e. less than one week, one week, between one week and one month, and more than one month). Lastly, participants were asked: ‘Why did you return to using electronic nicotine products?’. Responses included: I missed it; I couldn’t handle the stress in my life without it; I don’t know, I just started using electronic nicotine products again without thinking about it; other people in my life use electronic nicotine products and it was too hard to quit around them; the physical symptoms of withdrawal were too much to take.

Intolerance for Smoking Abstinence Questionnaire (IDSQ) is a 17-item measure assessing intolerance of symptoms associated with nicotine abstinence ^24^. For the current study, we used a measure that was adapted from the original IDSQ scale to assess intolerance of abstinence from using e-cigarettes (vaping; IDQV) ^25^. Items are rated on a Likert-type scale from 1 (strongly disagree) to 5 (strongly agree). A total score represents high intolerance for abstinence (α=0.91).

Motives were measured using the Wisconsin Inventory of Smoking/Vaping Dependence Motives-Brief (eWISDM-Brief) ^26-28^. The 37-item eWISDM-Brief measures motivations for peoples’ use of e-cigarettes. It was developed originally for smoking^28^ and was adapted to e-cigarette use ^26,27^. For the current study, it demonstrated strong internal consistency (α=0.96). Responses are measured on a seven-point Likert-type scale from 1 to 7 (not at all like me to very much like me). Averages of subscales [affiliative attachment (α=0.88), automatic habit (α=0.92), loss of control (α=0.90), cognitive enhancement (α=0.92), craving (α=0.89), cue exposure (α=0.72), emotional enhancement (α=0.88), social (α=0.93), taste and sensory (α=0.89), tolerance (α=0.90), and weight control (α=0.87)] were calculated and a total score was summed from these subscales; total scores ranged from 11 to 77.

Distress intolerance was measured using the Discomfort Intolerance Scale (DIS)^29^, the Distress Tolerance Scale (DTS) ^30^, and the Tolerance of Negative Affective States (TNAS) ^31^. The DIS uses seven items to assess tolerance of uncomfortable physical sensations (α=0.66). The measure uses a six-point Likert-type scale that ranges from 1 (not at all like me) to 6 (very much like me); total scores are summed (ranging from 7 to 42), with higher scores reflecting greater ability to tolerate distress. The DTS measures tolerance of negative emotional states. Responses range from 1 (strongly agree) to 5 (strongly disagree), and the average of the 15 items is calculated. Higher scores reflect greater ability to tolerate negative emotional states. The Tolerance of Negative Affective States (TNAS) uses 21 items to measure tolerance of specific emotions including: fear–distress, sadness–depression, anger, disgust, anxious–apprehension, and negative social emotions. An overall TNAS score measures tolerance of negative affective states comprehensively. Responses are scored using a Likert scale of 1 (very intolerant) to 5 (very tolerant).

### Data analytic strategy

Overall, 595 (98.8%) participants completed all variables, and three participants were removed who were aged >29 years at the time of the study, bringing the total sample size of the survey to 592. After reporting frequencies of all participants, we examined the differences between motivations, abstinence experiences, and distress intolerance between those who had and had not tried to quit e-cigarettes using t-tests. We also used ANOVAs to compare differences in the dependence on e-cigarettes based on quitting behaviors (i.e. number of times tried to quit or cut down, time since last quit attempt, length of quit attempt, and reason for quit attempt failure). Scheffe’s *post hoc* analysis was employed to compare between-group differences. Finally, three logistic regression models examined the association between the sociodemographic and behavioral variables and a quit attempt (Model 1), all previous variables and e-cigarette abstinence intolerance (Model 2), and all previous variables and motives to use e-cigarettes (using the eWISDM; Model 3).

### Question path

We employed a semi-structured interview to ask participants about their use and perceptions of e-cigarettes while allowing the researcher to probe deeper for greater understanding. The question path was developed alongside the quantitative items to allow responses from the interviews to explain survey findings. For some questions, participants were provided with images of survey findings and asked to explain their thoughts. For example, participants were prompted with ‘73% of survey respondents reported they had tried to quit or cut down e-cigarette use at some point. How did you answer this question?’. If the participant said they answered ‘no’, they were asked ‘Do you know of anyone who has tried to quit using an e-cigarette? Tell me what you know about their experience’. Participants that answered ‘yes’ were prompted with ‘Tell me about that’. Additional questions asked were: ‘What might be some reasons you or other young adults would try to quit using e-cigarettes?’, and ‘What might be challenging for you if you tried to quit using e-cigarettes today?’.

### Qualitative data analysis

Using NVivo 11, two trained coders coded all transcripts. Intercoder reliability ranged from a kappa (κ) 0.53 to 1.00 (mean=0.98, SD=0.09). All but one of the 25 transcripts were found to have a κ>0.90, and all discrepancies were discussed until consensus was met. Next, all authors conducted a thematic analysis using a qualitative analysis framework ^32^. To do this, we employed an iterative process of reading the coded transcripts and identify re-occurring patterns. We then discussed themes until consensus was met. Representative quotes were identified for themes and to represent quantitative findings.

### Mixed-methods data analysis

The point of convergence was at the point of analysis, allowing the themes to organize quantitative findings into meaningful sections. Further, representative quotes provided explanations in the participants’ language that helped explain survey findings. By initially collecting quantitative data and then interviewing a sub-sample of these participants, this study used qualitative data to provide deeper interpretation of the quantitative findings.

## RESULTS

### Sociodemographics

Overall, the final sample of survey responses (n=592) included mainly White (75.5%) females (67.2%) (Table 1). Some level of e-cigarette dependence was found among 86.8% (n=514) of participants, and 73.5% had tried to quit using e-cigarettes. The final sub-sample of those who completed an interview (n=25) included mainly White (72.0%) males (68.0%) who had some level of dependence on e-cigarettes (84.0%). Among these interviewees, 64.0% had tried to quit using e-cigarettes (additional participant information is given in Table 1).

**Table 1:** Sociodemographic characteristics of past 30-day e-cigarette users, from a cross-sectional survey (N=592) and interview (N=25) in 2021

*Characteristics*	*Survey* *(N=592)*		*Interview* *(N=25)*	
	*n*	*%*	*n*	*%*
**Gender**				
Male	166	28.0	17	68.0
Female	398	67.2	8	32.0
Non-binary/other	28	4.8		
**Race/ethnicity**				
White	447	75.5	18	72.0
African-American	40	6.8	3	12.0
Asian/Asian American	28	4.7	1	4.0
Hispanic/Latino	40	6.8	3	12.0
Biracial/multi-racial/other	37	6.2		
**Age** (years)				
18–20	124	20.9	6	24.0
21–24	285	48.2	10	40.0
25–29	183	30.9	9	36.0
**Education level**				
High school diploma or lower/trade	110	18.5	2	8.0
Some college	275	46.5	16	64.0
Bachelor’s degree or higher	207	35.0	7	28.0
**Marital status**				
Single	536	90.5	22	88.0
Married	56	9.5	3	12.0
**Smoker status**				
Dual e-cigarette user and smoker	184	31.1	10	40.0
Past smoker	342	57.8	14	56.0
Never smoker*	66	11.1	1	4.0
**E-cigarette dependence**				
No	78	13.2	4	16.0
Low	160	27.0	2	8.0
Moderate	165	27.9	6	24.0
High	189	31.9	13	52.0
**E-cigarette use during COVID-19**				
Increased	312	52.7	16.0	64.0
Decreased	69	11.7	1.0	4.0
Stable	211	35.6	8.0	32.0
**Tried to quit vaping**				
Yes	435	73.5	16	64.0
No	157	26.5	9	36.0
**PHQ-2,** mean (SD)	4.98 (1.99)		5.0 (2.08)	
**GAD,** mean (SD)	4.38 (1.81)		5.64 (2.16)	

* Missing responses from those who had never tried a cigarette: n=66 survey, n=1 interview.

PHQ-2: two-item Patient Health Questionnaire; scores range 2–8, higher scores reflect greater depression symptoms. GAD-2: two-item Generalized Anxiety Disorder; scores range 2–8, higher scores reflect greater worry and general anxiety symptoms.

Themes that derived from the qualitative interviews included: social, emotional, and physical distress as reasons for failed e-cigarette quit attempts.

### Emotional motivations to quit e-cigarettes

When asked about tolerance of negative emotions using the TNAS, those who had tried to quit using e-cigarettes reported greater tolerance of anger than those who had not (t[590]=1.75, p=0.041). There were no other significant differences found between the emotional regulation of those who had and had not tried to quit using e-cigarettes. Participants’ overall discomfort intolerance and distress intolerance were not significantly different. Interview participants described difficulty quitting due to emotional distress. Emotional distress that participants described included overcoming stressful events present during emerging adulthood (e.g. stress from school) and relational stress (e.g. parents’ divorce, breaking up with significant other):

*‘Something bad will happen to my family or something bad will happen with my finances or my school, and then it’ll just be too much and push me over the edge and I’ll just get right back on it.’* (Participant 4)

### Social motivations to quit e-cigarettes

Using the eWISDM score, we found that those who had tried to quit using e-cigarettes reported higher scores for affiliative attachment (t[291.6]=2.52, p=0.006) and social reasons (t[258.5]=3.04, p=0.002) than those who had not tried to quit. Social influences that hindered the quitting process included attending parties where alcohol and e-cigarettes were present:

*‘The social aspect of being around people that vape is really hard, because my boyfriend [and I] will be sitting in bed together on our phones and then he’ll randomly pull his vape out and the smoke hits me and it reminds me of what I could be having.’* (Participant 21)

### Physical motivations to quit e-cigarettes

Loss of control, craving, cue exposure, tolerance of withdrawal, and weight control was reported greater among those who had tried to quit compared to those who had not (all p<0.001). Interview participants described difficulty quitting due to craving and the withdrawal symptoms, such as getting sick, when they tried to quit:

*‘Cravings ... When you quit you get sick. Just being jittery and super angry the whole time, and I finally just broke.’* (Participant 2)

When asked about their ability to tolerate distress during periods of abstinence (using the IDQV) participants who had tried to quit using e-cigarettes were found to have a significantly greater intolerance for vaping abstinence than those who had not tried to quit (t[590]=2.85, p=002).

### E-cigarette dependence and quitting e-cigarettes

We also examined differences in e-cigarette dependence based on quitting behavior. Participants who had tried to quit or cut down on e-cigarettes three or more times (mean=10.88, SD=4.6) had a significantly greater dependence on e-cigarettes than those who had only tried once (mean=8.69, SD=5.0; p=0.002) (Table 3). Those whose last quit attempt lasted less than a week (mean=12.99, SD=3.7) were significantly more dependent on e-cigarettes than those who quit for one week (mean=9.72, SD=4.0; p<0.001), between one week and one month (mean=9.36, SD=4.4; p<0.001), and more than a month (mean=7.43, SD=4.9; p<0.001). Those whose quit attempt lasted more than one month (mean=7.43, SD=4.9) were significantly less dependent than those whose attempt lasted one week (mean=9.72, SD=4.0; p=0.005) and between one week and one month (mean=9.36, SD=4.4; p=0.012). When asked about the experience of quitting, one participant explained:

**Table 2:** Differences between motivations, abstinence experiences, and stress among e-cigarette users ever quit attempt, from a cross-sectional survey (N=592) and follow-up interview (N=25) in 2021

	*Have tried to quit e-cigarettes*		*p*	*Interview quotes*
	*Yes*	*No*		
	*N=435*	*N=157*		
**eWISDM**				
Affiliative attachment*	2.78 (1.72)	2.40 (1.62)	0.006	*‘I’m so used to just this act of smoking. Once I hit it for the day it’s on, I’m hitting it every five minutes, if not more. I’m just hitting, hitting it, hitting it, hitting.’* (Participant 17) *‘They [challenges of quitting] could make me super irritable. I don’t know about anxious, grumpy, irritable, probably hungry or something.’* (Participant 23)
Automatic habit*	5.11 (1.58)	4.47 (1.96)	0.001	
Loss of control*	3.90 (1.76)	3.01 (1.65)	0.001	
Cognitive enhancement	4.03 (1.86)	3.77 (1.90)	0.068	
Craving*	4.29 (1.70)	3.67 (1.87)	0.001	
Cue exposure	4.21 (1.55)	3.37 (1.52)	0.001	
Affective enhancement	4.22 (1.71)	3.99 (1.76)	0.076	
Social*	4.43 (1.83)	3.90 (1.97)	0.002	
Taste and sensory*	5.27 (1.50)	5.24 (1.63)	0.410	
Tolerance	4.27 (1.88)	3.62 (1.95)	0.001	
Weight control*	2.97 (1.81)	2.38 (1.59)	0.001	
Total*	45.49 (13.31)	39.82 (14.09)	0.001	
**IDQV**	2.91 (0.60)	2.75 (0.60)	0.002	*‘I quit once, once a couple of years ago and I did like a solid two weeks, and I remember the withdrawal symptoms were pretty, not great ... I was nauseous for a good day or two. The headaches was the worst part, I had a week-long headache. Migraines that I just could not get rid of, I was shaky as crap. I was moody.’* (Participant 13)
**TNAS**				
Sad	3.03 (1.04)	3.01 (1.07)	0.398	*‘It made me very irritable and angry and antsy, and I didn’t last half a day.’* (Participant 15) *‘Biggest challenge in quitting … Honestly, it’s really just the whole thing. It’s like if I don’t have it, I will freak out. It’s hard for me to do anything. I get angrier than I should.’* (Participant 14)
Anger	2.93 (1.07)	2.75 (1.11)	0.041	
Fear-distress	2.74 (0.93)	2.75 (0.91)	0.454	
Disgust*	3.16 (0.95)	3.08 (1.08)	0.210	
Anxiety apprehension	2.85 (0.91)	2.84 (0.86)	0.450	
Negative social emotions	2.66 (0.88)	2.69 (0.81)	0.334	
Total	2.87 (0.70)	2.83 (0.70)	0.315	
**DIS**	3.51 (0.86)	3.59 (0.83)	0.147	*‘I guess for a week, I was feeling a little fidgety ... the longing to have some but it was a really tough time.’* (Participant 10)
DTS*	2.98 (0.85)	2.98 (0.94)	0.496	*‘Basically, whenever mine runs out like just a general statement I can’t think of really anything specific, but whenever mine does run out and if I don’t have enough money for it, I kind of am like well shit the world’s over. Really genuinely, and I have depression.’* (Participant 1)

eWISDM: Wisconsin Inventory of E-Cigarette Dependence Motives-Brief; scores range 1–7 with higher scores indicating greater motives for e-cigarette use. TNAS: Tolerance of Negative Affective States; scores range 1–5 with higher scores indicating greater tolerance of each negative emotion). IDQV: Intolerance for Vaping Abstinence Withdrawal; scores range 1–5 with higher scores indicating greater intolerance for vaping abstinence. DTS: Distress Tolerance Scale; scores range 1–5 with higher scores indicating greater intolerance for negative feelings. DIS: Discomfort Intolerance Scale; scores range 7–42 with higher scores indicating less able to tolerate uncomfortable physical sensations.

* Levene’s test of equal variances, equal variances not assumed.

**Table 3:** E-cigarette dependence and e-cigarette quitting behaviors among a sample of past 30-day e-cigarette users with at least one quit attempt, from a cross-sectional survey (N=435) and follow-up interview (N=16) in 2021

*Response*	*Total* *(N=435)*	*Penn State E-cigarette Dependence*	*F*	*p*	*Interviewed* *(N=16)*	*Interview quotes*
	*n (%)*	*Mean (SD)*			*n (%)*	
**Number of times tried to quit or cut down**						
1	80 (18.4)	8.69 (5.0)^a^	7.19	0.001	1 (6.3)	*‘I tried to cut down like in the beginning of my junior year when I started buying my own, and that was mostly because I didn’t want to keep having to buy them every weekend ... I predict that when I graduate college, I would like to stop using it.’* (Participant 18)
2	118 (27.1)	9.74 (4.6)			3 (18.7)	*‘I’ve tried a couple of times, but they only last a good day or two, so I don’t even know if they can be full quit attempts. I put it in my mind that I was going to cut down – the last time I did that was New Years. I made a resolution that I’m not going to vape as much, and I broke it so …’* (Participant 13)
≥3	237 (54.5)	10.88 (4.6)^b^			12 (75.0)	*‘I’ve tried to quit using [e-cigarettes] too many times to count at this point … You wake up in the morning, and it’s the first thing on my mind. I wake up and reach for it, and I don’t have it and it’s just this constant buzz almost in the back of your head telling you to reach for it, telling you to take that hit.’* (Participant 14)
**Time since last quit attempt**						
Within last 30 days	136 (31.3)	10.50 (4.8)	1.73	0.178	10 (62.5)	*‘I have tried to quit multiple times and try to cut down multiple times. I would say that my quitting phase that I’m in right now has been the strongest because I haven’t been as focused on vaping.’* (Participant 11)
Within last 6 months	186 (42.8)	9.68 (4.7)			5 (31.2)	*‘The ways I tried to quit was the nicotine gum and the patches, but I’d still go back and hit other peoples’ vapes.’* (Participant 1)
More than 6 months ago	113 (25.9)	10.58 (4.8)			1 (6.3)	*‘I lowered my nicotine amount slowly over the course of a couple months, and then you just stop craving it. I’ve done it before to a certain extent, not entirely quit but definitely cut down.’* (Participant 20)
**Length of quit attempt**								
<1 week	148 (34.0)	12.99 (3.7)^d,e,f^	38.56	0.001	9 (56.2)	*‘Yeah, I’ve done it a few times. It’s pretty hard like the first two or three days and then you’re in the clear, but if I have a drink or a couple of drinks, I really want one.’* (Participant 3)	
1 week	74 (17.0)	9.72 (4.0)^c,f^			2 (12.5)	*‘It’s the withdrawal symptoms are really tough because it’s almost like your whole body is just like does not have any energy. I’ll wake up and then I’ll just not feel like getting out of bed for the entire day and then you get headaches, and you start shaking a bunch and all that stuff and it just makes it really difficult.’* (Participant 4)	
>1 week and ≤1 month	103 (23.7)	9.36 (4.4)^c,f^			5 (31.3)	*‘I throw it away on Mondays … if I didn’t drink, I could quit them no problem.’* (Participant 25)	
>1 month	110 (25.3)	7.43 (4.9)^c,d,e^			0 (0.0)		
**Reason for quit attempt failure**								
Missed it	85 (19.5)	10.47 (4.4)^i^	15.96	0.001	2 (12.5)	*‘Not having something to do with your hands and mouth like just doing something I know that the easy fix is just getting up and go do something, you know but um I would say that’s probably the biggest challenge.’* (Participant 19)	
Couldn’t handle stress	100 (23.0)	12.52 (4.0)^i,j^			7 (43.8)	*‘Also, just stress and stuff when things get to be a bit too much it’s a really good stress reliever for me so I, I think it is but um yeah just not having that crutch is really hard too.’* (Participant 21)	
Restarted without thinking	89 (20.5)	7.83 (5.3)^g,h,j,k^			0 (0.0)		
Hard to quit around others	104 (23.9)	9.30 (4.2)^h,k^			4 (25.0)	*‘I definitely have tried to stop before. I feel like the social aspects, always kind of like brings me back in because there’s always somebody with something who’s always offering, you know, people to hit it.’* (Participant 5)	
Physical symptoms of withdrawal	34 (7.8)	13.00 (3.3)^i,j^			1 (6.2)	*‘Not nauseous per say but like, headaches, like, I felt like I had a fever or something or could like, started feeling like really bad.’* (Participant 10)	
Other	23 (5.3)	7.61 (4.2)			2 (12.5)	*‘I have tried. The thing is that it’s, just something like trying to kick a habit that’s really hard to kick because it’s due to the convenience and the cravings that you get afterwards.’* (Participant 22)	

Penn State E-Cigarette Dependence scale range 0–20 with scores closer to 0 indicating less dependence on e-cigarettes and scores closer to 20 indicating more dependence on e-cigarettes; α=0.05.

a Significantly different than ‘≥3’.

b Significantly different than ‘1’.

c Significantly different than ‘<1 week’.

d Significantly different than ‘1 week’.

e Significantly different than ‘>1 week and ≤1 month’.

f Significant different than ‘>1 month’.

g Significantly different than ‘Missed it’.

h Significantly different than ‘Couldn’t handle stress’.

i Significantly different than ‘Restarted without thinking’.

j Significantly different than ‘Hard to quit around others’.

k Significantly different than ‘Physical symptoms of withdrawal’.

*‘I think it’s pretty akin to quitting drinking caffeine, or anything else. When your body develops a neuro chemical dependence on something it’s not easy, regardless of what it is you’re trying to quit.’* (Participant 16)

Those who restarted e-cigarette use without thinking were significantly less dependent than those who restarted because they missed it (mean=7.83, SD=5.3; p=0.009) and those who restarted because they could not handle the stress (mean=12.52, SD=4.0; p<0.001). Also, those who restarted due to stress were more dependent than those who restarted because others in their life used e-cigarettes (p<0.001). When asked if they had ever tried to quit using e-cigarettes, one interview participant stated:

*‘I thought about it, but you haven’t actually made any efforts to quit it, because I mainly use it to relieve stress, or just have a quick pause and something that I’m doing and I just haven’t found a replacement for it.’* (Participant 8)

Those who restarted due to physical symptoms of withdrawal were more dependent than those who restarted without thinking about it (mean=13.00, SD=3.3; p<0.001). During the interviews, one participant explained that the *‘withdrawal from nicotine’* (Participant 23) was likely the biggest challenge of quitting e-cigarettes. No significant associations were found between time since the last quit attempt and participants’ Penn State E-Cigarette Dependence scores. Table 3 gives the scores and representative quotes from interview participants.

### Attempting to quit e-cigarettes

In Model 1, we examined the unique contribution of the sociodemographic and behavioral questions while controlling for covariates. Age, education level, e-cigarette dependence, and use during COVID-19 were significantly associated with at least one past e-cigarette quit attempt. Increased age was associated with lower odds of a quit attempt (AOR=0.92; 95% CI: 0.86–0.99) (Table 4). Those who had completed a Bachelor’s degree or higher (AOR=1.91; 95% CI: 1.08, 3.41) had higher odds of a past e-cigarette quit attempt compared to those with a high school diploma or lower. Those with moderate (AOR=3.14; 95% CI: 1.65–5.99) or high dependence (AOR=3.00; 95% CI: 1.57–5.72) on e-cigarettes reported greater odds of an e-cigarette quit attempt than those with no e-cigarette dependence. Further, those who decreased their use of e-cigarettes during COVID-19 were 2.39 times (95% CI: 1.14–4.99) as likely to have attempted to quit e-cigarettes as those who increased during COVID-19.

**Table 4:** Factors associated with attempting to quit e-cigarettes among a sample of past 30-day e-cigarette users, from a cross-sectional survey (N=592) in 2021

*Variables*	*Model 1*	*Model 2*	*Model 3*
	*AOR (95% CI)*	*AOR (95% CI)*	*AOR (95% CI)*
**Gender**			
Male ®	1	1	1
Female	1.47 (0.94–2.32)	1.46 (0.91–2.35)	1.20 (0.72–1.98)
Non-binary/other	1.37 (0.50–3.80)	1.53 (0.53–4.42)	1.59 (0.52–4.82)
**Race/ethnicity**			
Non-Hispanic White ®	1	1	1
African American/Black	0.92 (0.44–1.94)	0.99 (0.46–2.16)	1.05 (0.47–2.34)
Asian	1.16 (0.45–2.97)	1.43 (0.52–3.95)	1.69 (0.59–4.82)
Hispanic	1.26 (0.58–2.73)	1.48 (0.64–3.40)	1.72 (0.74–3.97)
Biracial/Other	1.02 (0.45–2.35)	0.96 (0.40–2.29)	1.00 (0.41–2.46)
**Age** (years)	0.92 (0.86–0.99)	0.92 (0.85–0.99)	0.92 (0.85–1.00)
**Education level**			
High school diploma or lower ®	1	1	1
Some college	1.60 (0.95–2.70)	1.71 (0.98–2.98)	1.62 (0.91–2.90)
Bachelor’s degree or higher	1.91 (1.08–3.41)	1.84 (1.01–3.36)	1.69 (0.90–3.19)
**Marital status**			
Single ®	1	1	1
Married	0.64 (0.34–1.21)	0.54 (0.28–1.05)	0.50 (0.25–1.00)
**Smoker status**			
Dual user ®	1	1	1
Past smoker	1.49 (0.96–2.32)	1.60 (1.01–2.53)	1.73 (1.06–2.81)
Never smoker	1.13 (0.56–2.27)	1.17 (0.57–2.41)	1.22 (0.57–2.61)
**E-cigarette dependence**			
No ®	1	1	1
Low	1.80 (0.98–3.32)	1.25 (0.64–2.53)	1.29 (0.63–2.62)
Moderate	3.14 (1.65–5.99)	1.44 (0.68–3.04)	1.49 (0.60–3.67)
High	3.00 (1.57–5.72)	1.15 (1.51–2.63)	1.38 (0.46–4.15)
**E-cigarette use during COVID-19**			
Increased ®	1	1	1
Decreased	2.39 (1.14–4.99)	2.49 (1.14–5.47)	2.15 (0.96–4.80)
Stable	0.79 (0.52–1.21)	0.86 (0.55–1.34)	0.91 (0.58–1.44)
**PHQ-2**	1.04 (0.90–1.19)	1.03 (0.89–1.19)	1.00 (0.86–1.16)
**GAD-2**	1.06 (0.93–1.21)	1.03 (0.90–1.18)	1.02 (0.89–1.17)
**IDQV**			
Withdrawal		1.41 (1.03–1.93)	1.46 (0.95–2.24)
Cognitive coping		0.53 (0.40–0.70)	0.56 (0.42–0.75)
**eWISDM**			
Affiliative attachment			0.88 (0.73–1.06)
Automatic habit			1.04 (0.86–1.27)
Loss of control			1.45 (1.08–1.94)
Cognitive enhancement			0.97 (0.83–1.14)
Craving			0.72 (0.52–1.00)
Cue exposure			1.40 (1.12–1.76)
Emotional enhancement			0.80 (0.66–0.98)
Social			1.01 (0.89–1.15)
Taste and sensory processes			0.98 (0.83–1.15)
Tolerance			0.94 (0.73–1.20)
Weight control			1.13 (0.97–1.31)
R^2^	0.137	0.218	0.276

AOR: adjusted odd ratio. Model 1: included sociodemographic information and behavior questions. Model 2: as in Model 1 plus the Intolerance for Vaping Abstinence (IDQV) scale which measured withdrawal and coping intolerance (total score). Model 3: as in Model 2 plus the Wisconsin Inventory of Smoking/Vaping Dependence Motives-Brief (eWISDM-Brief) that measured different motivations for use. ® Reference categories.

Next, we included measures of e-cigarette abstinence by including the two subscales from the IDQV. We found that those who experienced greater intolerance of withdrawal symptoms had increased odds of attempting to quit (AOR=1.41; 95% CI: 1.03–1.93) while greater lack of cognitive coping decreased participants’ odds of attempting to quit (AOR=0.53; 95% CI: 0.40–0.70). In this Model 2, age, education level, past smoker status (compared to dual user), and decreased use during COVID-19 (compared to increased use) were also associated with a quit attempt.

In Model 3, all variables were included as well as the subscales of the eWISDM measure. When controlling for all covariates, loss of control (AOR=1.45; 95% CI: 1.08–1.94) and cue exposure (AOR=1.40; 95% CI: 1.12–1.76) were associated with increased odds of attempting to quit and those motivated by emotional enhancement had lower odds of a quit attempt (AOR=0.80; 95% CI: 0.66–0.98). Moreover, those who were past smokers had increased odds of a quit attempt (AOR=1.73; 95% CI: 1.06–2.81) and lack of cognitive coping decreased participants’ odds of a quit attempt (AOR=0.56; 95% CI: 0.42–0.75). Full results from the three models are given in Table 4.

## DISCUSSION

Similar to prior work ^8^, we found more than half of our participants intended to quit using e-cigarettes. Prior research suggests that dependence, harm perceptions, and social influence are associated with attempting to quit vaping, with harm perceptions of e-cigarettes found to be the most influential factor on someone’s serious attempt to quit vaping ^8,33^. Although our study did not examine harm perceptions, e-cigarette users discussed adverse social, emotional, and physical experiences when attempting to quit using e-cigarettes. Using this insight, we returned to the quantitative data, where we discovered cue exposure and loss of control increased the odds of a quit attempt while those who were motivated for use by emotional enhancement were less likely to have attempted to quit. Thus, understanding and avoiding cues and learning how to maintain control may be helpful cessation strategies, and those developing cessation programs should consider helping e-cigarette users find other alternative emotional enhancements to prevent relapse.

Similar to prior research that found dual users emphasized quitting all tobacco and nicotine products ^14^, we found past smokers were more likely than dual users to have attempted to quit using e-cigarettes. Although this past smoker definition may include those who were not daily smokers, it is possible that past users who have already quit smoking intend to quit all nicotine-containing products. Although we did not find distress or discomfort intolerance to be associated with a quit attempt, we did find that a lack of cognitive coping skills decreased odds of attempted quitting. Thus, focusing cessation programs on cognitive coping strategies could help address daily stressors and withdrawal symptoms identified in prior research ^34^.

We found e-cigarette dependence to be associated with reporting relapse due to stress and physical symptoms of withdrawal. Given these findings, along with findings that emotion intolerance was associated with a quit attempt, it is possible that distress intolerance may be associated with e-cigarette cessation relapse similarly to smoking cessation to relapse ^18^. E-cigarette cessation programs should consider adapting anxiety-reducing treatment methods consistent with smoking cessation programs ^35^. Further, future research should examine efficacy of such programs using longitudinal and ecological momentary assessment designs to examine dose response for distress intolerance and e-cigarette quit attempts.

### Limitations

Due to the cross-sectional study design and non-random sampling methods, no causal inferences can be made about the broader young adult population. Given the self-reported nature of the study, data are subject to response bias and recall bias. We also had some groups that were predominantly represented (e.g. females) than other groups in the survey; however, the interview participants were predominantly made up of males. Thus, these samples may not have been similar enough to draw conclusions from the findings, and they lack generalizability to diverse populations. Further, those who had already successfully quit were not included in the study, which may have influenced findings about dependence and distress tolerance, which those who experience greater distress tolerance being able to quit and not qualify for the study. Given the volatility of e-cigarettes, it is possible that products included in our analysis may not be sold in current and future markets. Finally, although we controlled covariates, data interpretation is limited by residual confounding.

## CONCLUSIONS

Young adults’ experiences attempting to quit e-cigarettes vary widely. Young adult e-cigarette users, particularly those with higher levels of dependence, may have already experienced several unsuccessful quit attempts when trying to quit using e-cigarettes. Those working in prevention on college campuses and industries with entry-level employees may need to provide cessation services to young audiences who want to quit using e-cigarettes. Helping these young e-cigarette users develop stronger cognitive coping skills and addressing other factors that motivate their use may encourage those who do not think they can successfully quit using e-cigarettes to make an attempt.

## Data Availability

The data supporting this research are available from the authors on reasonable request.
